# Association between child ADHD and caregivers’ mental health and health-related quality of life: results from a population-based study

**DOI:** 10.1007/s11136-026-04244-6

**Published:** 2026-05-03

**Authors:** Ha N.D Le, Roma Yee, Sithara Wanni Arachchige Dona, Tim Silk

**Affiliations:** 1 Deakin Health Economics, School of Health and Social Development, Deakin Institute for Health, Deakin University, 221 Burwood Hwy, Burwood, Geelong, VIC 3025 Australia; 2 School of Psychology, Deakin University, Geelong, Australia

**Keywords:** HRQoL, ADHD, Children, Caregiver, Mental health

## Abstract

**Purpose:**

To explore (1) the association between child attention-deficit/hyperactivity disorder (ADHD)/ADHD symptoms and their caregivers’ health-related quality of life (HRQoL)/HRQoL dimensions and their mental health, and (2) the factors that may influence caregivers’ HRQoL.

**Methods:**

Data were drawn from the population-based Longitudinal Study of Australian Children (LSAC) at the cross-sectional Child Health CheckPoint data collection. ADHD was parent-reported of the child’s ADHD diagnosis at 10–11 years. ADHD symptoms were measured using the Strengths and Difficulties inattention-hyperactivity subscale (e.g. score > 8). Caregivers’ HRQoL was measured using the Assessment of Quality of Life 8 Dimensions (AQoL-8D) at children aged 11–12 years. Caregivers’ mental health was measured using the Kessler 6. Multivariable linear regression analysis was used to estimate the association between children’s ADHD/ADHD symptoms and caregivers’ HRQoL and mental health.

**Results:**

Child ADHD was associated with poorer caregivers’ mental health (mean difference = – 1.310, 95% CI − 2.439, − 0.181). Caregiver mental health attenuated the positive association between child ADHD and caregiver HRQoL (mean difference = 0.049, 95% CI − 0.008, 0.107). Child behavioural issues and financial hardship were associated with lower caregivers’ HRQoL, while high relationship quality or level of education was associated with better HRQoL.

**Conclusion:**

This study confirms the association between child ADHD and poor caregivers’ mental health, but not caregivers’ HRQoL, once caregivers’ mental health was accounted for. Child behavioural issues and financial hardship were associated with reduced caregivers’ HRQoL. Future policy or service development for families of children with ADHD may consider these factors in tandem with ADHD.

## Introduction

Attention-deficit/hyperactivity disorder (ADHD) is a neurodevelopmental condition characterised by persistent and developmentally inappropriate symptoms of inattention and/or hyperactivity-impulsivity, which substantially disrupt the child’s functioning across various settings such as home, school, and social environments [1]. ADHD is commonly diagnosed during childhood or adolescence and is frequently associated with a diverse and complex clinical presentation, often accompanied by overlapping comorbid conditions such as anxiety, depression and autism [2]. Prevalence estimates vary, ranging from 3.4 to 14%, with a global pooled prevalence of 8%, and boys are twice as likely as girls to be diagnosed (10% vs. 5%) [1].

Primary caregivers play an integral role in supporting children with ADHD, including navigating care systems and advocating for services, and the emotional and psychological demands of caregiving can be substantial [3, 4]. From a stress-process perspective, these ongoing caregiving demands represent chronic stressors that may strain caregivers’ psychological and social resources [5]. Consistent with this framework, caregivers of children with neurodevelopmental conditions report heightened levels of stress, psychological dysfunction, social strain, and health problems, as well as increased use of ineffective parenting strategies compared to parents of neurotypical children [6–8]. This can negatively influence not only caregivers’ mental health and well-being but also children’s outcomes.

Health-Related Quality of Life (HRQoL) has emerged as an important construct in measuring individual well-being [9]. HRQoL captures the physical, mental, and social aspects of health as perceived by individuals [10]. Based on Lazarus and Folkman’s stress and coping theory, HRQoL can be conceptualised as an outcome of sustained stress exposure and coping effectiveness over time [5], rather than as a direct consequence of child ADHD diagnosis alone. Despite its increasing importance, there is limited consensus on how to conceptualise and define HRQoL or on the optimal measurement approach [11, 12]. Variability in HRQoL tools and proxy versus self-report methods has limited comparability across studies [9, 13, 14].

While extensive literature has investigated the impact of ADHD on children’s HRQoL [15–18], far less is known about its impact on caregivers’ HRQoL or mental health. The most recent synthesised evidence comes from a broader review of caregiver outcomes in various developmental disorders, which included but did not isolate ADHD-related effects [3]. While studies on caregivers’ HRQoL are scarce, Klassen et al. (2008) suggested that child ADHD symptoms significantly affect caregivers’ emotional well-being and overall family functioning [19]. Meta-analytic and cross-sectional studies indicate that parenting stress may play a central role in linking child ADHD symptoms to caregiver well-being, consistent with both stress-process and family systems frameworks [20, 21]. Emerging evidence further highlights the importance of coping strategies, self-efficacy, and psychological resources in buffering the impact of caregiving stress on mental health and quality of life [22, 23]. Fernandes et al. (2015) suggested that parenting stress, not child ADHD symptoms directly, is more strongly related to caregiver HRQoL. These theoretical frameworks suggest that the association between child ADHD and caregiver HRQoL is likely indirect and multifactorial, shaped by caregiving stress, coping processes, caregiver mental health, and other factors. The most common significant predictors of caregivers’ HRQoL included child ADHD symptom severity, socio-economic status, educational status, marital status, child co-morbidities and caregiver mental health status (e.g., depression or anxiety) [24–27]. However, population-based evidence examining these relationships remains limited within the Australian context.

Given the paucity of research exploring the impact of child ADHD on caregivers’ mental health and wellbeing, understanding this association is critical to informing evidence-based policy and designing targeted interventions. Strengthening caregiver well-being may also yield reciprocal benefits for children with ADHD and contribute to improved overall family functioning. Drawing data from the Longitudinal Study of Australian Children (LSAC), we aim to explore: (1) the association between child ADHD or ADHD symptoms and caregivers’ HRQoL; (2) which domains are most affected by child ADHD or ADHD symptoms; (3) the association between child ADHD or ADHD symptoms and caregiver mental health; and (4) predictive factors influencing caregivers’ HRQoL.

## Methods

### Study design, participants, setting

LSAC is a nationally representative, longitudinal study that tracked the development of 10,000 children and families across Australia [28]. Details of the initial study design, methodology, recruitment and data collection have been recorded elsewhere [28, 29]. LSAC commenced in 2004 with two cohorts of 5000 children each, aged 0–1 (Birth, B cohort, N = 5107) and 4–5 (Kindergarten, K cohort, N = 4985) [30]. Data were collected biennially, with wave 10 being the latest data release in 2025. Study participants were both the child and their caregivers [28]. The Child Health CheckPoint dataset was a one-off cross-sectional bio-physical data collection conducted between waves 6 and 7 for consenting primary caregivers (parent 1) and their children from the B cohort at 11 to 12 years old/wave 6.5 (N = 1,874) [31]. As the Child Health CheckPoint dataset was the only wave where caregivers’ HRQoL data were collected, this study included children and caregivers from the Child Health CheckPoint data collection. Due to limited child and family data in the Child Health CheckPoint, relevant variables were sourced from LSAC wave 6 (age 10–11; N = 3,764). These data were linked with CheckPoint data for the analyses in this study.

## Measures

### Caregivers’ health-related quality of life

Caregivers’ HRQoL was measured at the child’s age of 11–12 years (wave 6.5) using the 35-item Assessment of Quality of Life-8D (AQoL-8D). The AQoL-8D is a generic multi-attribute utility instrument (MAUI) for measuring HRQoL with eight domains: independent living, happiness, mental health, coping, relationships, self-worth, pain, and senses [32]. The AQoL8D has high predictive validity [32] and high reliability (Cronbach alpha: 0.96) [32, 33]. The AQoL-8D was selected for the LSAC adult cohort because it captures both physical and psychosocial dimensions of health, which is particularly important for assessing the quality of life of parents in the general population [32]. Its emphasis on psychosocial domains improves sensitivity to caregiver-specific impacts, though it may reduce comparability with instruments that prioritise physical health [32].

### Caregivers’ mental health

Caregivers’ mental health was measured using the 6-item Kessler 6 (K6) psychological distress scale at child aged 10–11 years (wave 6) [34]. The K6 captures current symptoms of non-specific psychological distress over the past 30 days. It has demonstrated good to excellent internal consistency (alpha = 0.78–0.90) and has been widely used in international studies in adult populations [35].

### ADHD

Child ADHD status was parent-reported on the question “Does your child have any of these ongoing problems? Attention Deficit Disorder (ADD)/ADHD” at wave 6 (child aged 10–11 years).

### ADHD clinical symptoms

Child ADHD clinical symptom was measured using the five-item parent-reported Strengths and Difficulties Questionnaire (SDQ) hyperactivity-inattention subscale, measured at wave 6 [36]. We defined ADHD clinical symptoms using the standard cut-off of ≥ 90th percentile (score ≥ 8, possible scores range from 0 to 10) as per previous literature [37]. Children scoring ≥ 90th percentile on the Hyperactivity-Inattention subscale are approximately 18 times more likely to meet the Diagnostic and Statistical Manual for Mental Disorders (4th ed.; DSM-IV; American Psychiatric Association, 1994) criteria for ADHD than other children [38]. The SDQ hyperactivity-inattention subscale has high internal consistency reliability (alpha coefficient of 0.74) [39].

### Child and family factors

Information about child and caregiver characteristics (measured at wave 6) is described in Table 5. These characteristics were considered a priori based on the stress process and family frameworks [5] and the literature [20, 21]. Child total difficulties were measured using the 25-item Strengths and Difficulties Questionnaire total score [SDQ; 36, 40]. The SDQ is a validated behavioural and emotional health screening questionnaire used to evaluate child and adolescent mental health problems [40]. A score of 0–13 is considered normal, 14–16 is slightly raised/lowered, 17–19 is considered high, and 20–40 is very high [41]. Child’s ‘other medical conditions’ is a composite variable composed of 8 common co-morbid conditions, including eczema, asthma, vision problems, hearing problems, frequent headaches, diabetes, epilepsy, and chronic fatigue.

Parents’ post-secondary educational status was measured using a composite variable combining the responses from two questions from the LSAC dataset. Socioeconomic position was selected from the CheckPoint dataset using the Socioeconomic Indexes for Areas (SEIFA) Disadvantage Quintiles [31]. SEIFA is an aggregate measure of the average economic profile of a given geographic location, widely used to report the socioeconomic status of groups in Australia [42, 43]. A financial hardship scale was designed for the LSAC study using items from previous studies: the Early Childhood Longitudinal Study, Birth Cohort [44], the Australian Bureau of Statistics Household Expenditure Survey [45] and the Life Chances longitudinal study [46]. Partner relationship quality was measured using the Hendrick relationship scale [47].

### Ethics

LSAC and the CheckPoint data collection were approved by the Australian Institute for Family Studies [48] and the Royal Children’s Hospital (Melbourne, Australia) Human Research Ethics Committee (ref: 33225D). Written consents were obtained from all participating caregivers and their children. An ethics exemption was approved for this secondary analysis study by the Deakin University ethics committee (2024/HE000420).

### Statistical analyses

All statistical analyses were performed using Stata 18 [49]. The LSAC wave 6, B cohort dataset and the Child Health CheckPoint datasets (wave 6.5) were merged according to participant identifier number. There were 1,874 total matched observations in the analytic sample. Nineteen participants in the sample were missing data identifying their children’s ADHD status (N = 6) or HRQoL data (N = 13) and were excluded from the analysis. Given the relatively small amount of missing data (< 10%) in both outcome and main exposure variables, multiple imputation is less likely to meaningfully improve estimation of their association [50]. Therefore, we present the complete case analysis with the analytic sample comprising 1855 participants with complete ADHD status and HRQoL data. As a sensitivity check, we applied multiple imputation by chained equations, using linear regression for continuous variables (children’s HRQoL and their domains), logistic regression for binary variables (ADHD/ADHD symptoms), and ordinal logistic regression for ordinal variables (caregiver’s education). Results were consistent with the complete case analysis, confirming the robustness of our findings. A descriptive analysis was conducted and summarised as means and standard deviations for continuous data and frequencies and percentages for categorical data. Although the data for HRQoL are slightly skewed, the normality assumption is not a prerequisite for large datasets [51]. Thus, multivariable linear regression was used to explore the association between childhood ADHD/ADHD clinical symptoms and caregivers’ HRQoL/mental health problems. We also conducted the analyses using generalised linear models (log link, gamma distribution), which showed similar results. For the convenience of interpretation, we chose to use multivariable linear regression. The CheckPoint sample weight was used to correct for sample disproportionality (e.g. slightly more advantaged and less likely to be from regional or remote area, an Indigenous or non-English speaking background than LSAC wave 1) and to compensate for attrition rates (37%) and nonresponse bias [31, 52].

Covariates were first informed by the stress-process and family systems frameworks [5] and prior evidence [20, 21] and refined using statistical criteria to ensure model parsimony and stability. Multicollinearity was assessed using Pearson correlations, and highly collinear variables were not included simultaneously. The cutoff for multicollinearity coefficients was determined at > 0.5 due to the potential impact on the study results [53]. Six variables were excluded due to moderate (> 0.5) to strong (≥ 0.7) correlation: hyperactivity-inattention subscale, caregiver mental health, taking ADHD medication, ‘happiness in relationship’, parental efficacy, and parental coping. Several of these variables, including caregiver mental health, happiness, parental efficacy and coping, also overlap conceptually with HRQoL dimensions measured by the AQoL-8D. Therefore, to avoid over-adjustment, these factors were not included in the primary analysis. Given its central role in the stress-process and family systems frameworks, caregiver mental health was examined separately in a sensitivity analysis to explore the extent to which observed HRQoL differences were statistically associated with caregiver psychological distress. The Stata vselect (best option) command was then used for variable selection to assist in optimal model selection based on the initial included explanatory variables [54], with child ADHD as a fixed predictor variable. Based on the Bayesian Information Criterion (BIC) from the vselect [55] and the principle of parsimony, five predictor variables were selected for the final model exploring caregiver HRQoL and child ADHD: relationship quality, the child’s total difficulties, child’s other medical conditions, financial hardship, and parental higher education. For the association between caregivers’ mental health and child ADHD, the covariates include relationship quality, the child’s total difficulties, child’s other medical conditions, financial hardship, and parental higher education.

A secondary analysis was conducted to explore the association between ADHD clinical symptoms [36] and caregivers’ HRQoL using the same set of covariates. However, due to the categorisation of ADHD clinical symptoms using the SDQ inattention-hyperactivity scale, we did not include SDQ total difficulties in the covariates but instead included the SDQ Conduct and Emotional Subscales to measure child internalising and externalising problems.

## Results

**Fig. 1:**
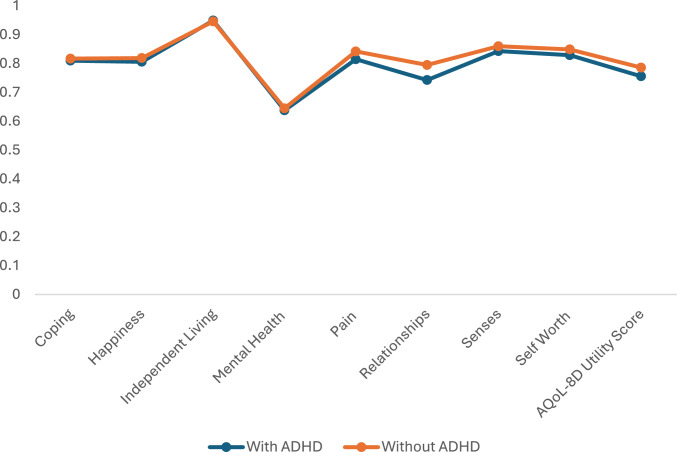
HRQoL and HRQoL dimensions of caregivers of children with and without ADHD

Selected characteristics of the analytical sample are presented in Table 1. Among children with parent-reported ADHD, 76% were male; about 60% met the threshold of ADHD clinical symptoms or other medical conditions; and 85% took ADHD medication. All caregivers in this sample were females. Compared to caregivers of children without ADHD, caregivers of children with ADHD had higher depression scale and hardship scale scores, but lower relationship quality and education level. Table 6 describes selected characteristics of children with and without ADHD clinical symptoms. We observed a slight difference in the relationship domain of HRQoL of caregivers of children with and without ADHD (Fig. 1).

**Table 1:** Selected child and family characteristics of the analytical sample at child aged 10–11 years

Characteristics	Child has ongoing ADHD		
	No	Yes	Total
	N = 1797	N = 58	N = 1855
Male, n (%)	851 (50.3%)	47 (75.8%)	898 (51.2%)
Child SDQ total difficulties score, mean (SD)	7.3 (5.1)	17.4 (6.7)	7.6 (5.6)
ADHD clinical symptoms	75 (4.5%)	35 (56.7%)	110 (6.3%)
Child’s other medical conditions	598 (35.3%)	37 (59.4%)	634 (36.2%)
Child taking ADD medication	0 (0.0%)	39 (84.9%)	39 (84.9%)
Parent 1 age, mean (SD)	42.1 (5.2)	41.8 (5.5)	42.1 (5.2)
Parent 1’s depression scale score, mean (SD)	9.3 (3.5)	10.1 (3.8)	9.3 (3.5)
Hendrick relationship quality scale, mean (SD)	4.3 (0.8)	4.2 (0.7)	4.2 (0.8)
Hardship scale, mean (SD)	0.3 (0.7)	0.3 (0.8)	0.3 (0.7)
Parent1 highest education level			
Postgraduate degree	176 (12.2%)	4 (8.4%)	181 (12.1%)
Graduate diploma/certificate	157 (10.9%)	3 (5.7%)	160 (10.7%)
Bachelor degree	353 (24.4%)	7 (12.5%)	359 (24.0%)
Advanced diploma/diploma, certificate, or other	760 (52.6%)	39 (73.4%)	798 (53.3%)

Continuous Variables: Mean (SD); Categorical Variables: n (%); Statistics computed using the Checkpoint survey weights; SDQ – Strengths and Difficulties Questionnaire; parent response

## Association between child ADHD/ADHD symptoms and caregivers’ HRQoL/HRQoL dimensions

In unadjusted analyses, we did not observe a statistically significant association between child ADHD and overall caregivers’ HRQoL at children aged 11–12 years (mean difference [MD] = − 0.031, 95% CI -0.008 to 0.026) (Table 2). When adjusted for child and family factors, we observed a positive association between parent-reported child ADHD and their HRQoL (MD = 0.076, 95% CI 0.026 to 0.112) (Table 3). While there is limited evidence on the minimally important difference (MID) for the AQoL-8D, this mean difference sits at the lower bound of the reported MIDs in published literature for AQoL of 0.06 utility points (95% CI 0.03 to 0.08) [56], suggesting a small but important difference in HRQoL.

**Table 2:** Association between caregivers’ overall HRQoL and child ADHD (unadjusted analysis)

Statistic	Whole analytic sample (N = 1855)	ADHD (N = 58)	No ADHD (N = 1797)	Unadjusted analysis	
	Mean (SD)	Mean (SD)	Mean (SD)	Mean difference	95% CI
Overall utility score	0.785 (0.004)	0.756 (0.190)	0.786 (0.152)	− 0.031	− 0.086, 0.026

Significance level at 5%; SD: Standard deviation; ADHD: Attention Deficit Hyperactivity Disorder; HRQoL: Health-related quality of life

**Table 3:** Association between caregivers’ overall HRQoL/HRQoL dimensions and child ADHD (adjusted analyses)

	Overall utility score		Coping		Happiness		Independent living		Mental health		Pain		Relationship		Senses		Self-worth	
Child ADHD	**0.076**	**0.016,0.135**	**0.069**	**0.026,0.112**	0.030	− 0.022,0.082	**0.032**	**0.004,0.060**	**0.056**	**0.016,0.097**	0.054	− 0.008,0.116	0.034	− 0.022,0.091	0.010	− 0.028,0.047	0.055	− 0.001,0.110
Caregiver relationship quality	**0.090**	**0.037,0.062**	**0.025**	**0.014,0.036**	**0.035**	**0.026,0.044**	**0.011**	**0.004,0.018**	**0.036**	**0.028,0.044**	**0.016**	**0.001,0.032**	**0.057**	**0.045,0.068**	**0.016**	**0.007,0.025**	**0.027**	**0.017,0.036**
SDQ total score	− **0.008**	− **0.010,-0.006**	− **0.006**	− **0.007,-0.004**	− **0.004**	− **0.005,-0.003**	− **0.002**	− **0.004,-0.001**	− **0.006**	− **0.007,-0.004**	− **0.006**	− **0.009,**− **0.004**	− **0.006**	− **0.008,**− **0.004**	− **0.002**	− **0.003,**− **0.001**	− **0.006**	− **0.007,**− **0.004**
Financial hardship	− **0.025**	− **0.045,-0.004**	− **0.018**	− **0.035,-0.001**	− **0.013**	− **0.030,0.004**	− **0.015**	− **0.028,-0.002**	− **0.019**	− **0.029,**− **0.008**	− 0.021	− 0.044,0.002	− 0.013	− 0.032,0.006	− 0.010	− 0.027,0.007	− 0.010	− 0.022,0.002
Other medical conditions	− 0.014	− 0.031,0.003	− 0.007	− 0.021,0.007	0.000	− 0.012,0.012	− **0.011**	− **0.022,**− **0.000**	0.002	− 0.011,0.016	− **0.026**	− **0.048,**− **0.004**	− 0.013	− 0.028,0.001	− 0.012	− 0.025,0.001	− 0.008	− 0.023,0.007
*Higher education, post-graduate as reference*																		
Graduate diploma/Certificate	− **0.025**	− **0.049,**− **0.001**	− 0.008	− 0.027,0.010	− 0.013	− 0.030,0.004	− 0.006	− 0.022,0.009	− 0.011	− 0.034,0.013	− 0.014	− 0.049,0.021	− 0.011	− 0.037,0.015	− **0.028**	− **0.051,**− **0.006**	− **0.026**	− **0.046,**− **0.005**
Bachelor degree	0.007	− 0.012,0.026	0.002	− 0.014,0.018	− 0.007	− 0.021,0.008	0.002	− 0.009,0.012	0.007	− 0.011,0.025	0.018	− 0.010,0.047	0.014	− 0.006,0.035	− 0.001	− 0.018,0.016	− 0.006	− 0.021,0.010
Advanced/diploma, certificate or other	− **0.026**	− **0.046,**− **0.007**	− 0.012	− 0.027,0.004	− 0.009	− 0.022,0.005	− **0.017**	− **0.030,**− **0.004**	− 0.003	− 0.021,0.015	− **0.038**	− **0.065,-0.012**	− 0.006	− 0.026,0.015	− 0.011	− 0.029,0.007	− **0.039**	− **0.056,-0.023**

SD: Standard deviation; CI: confidence interval; ADHD: Attention Deficit Hyperactivity Disorder; HRQoL: Health-related quality of life; Significance level at 5%; Statistically significant estimates in bold; N = 1855

Conversely, in unadjusted analysis, we found a negative association between child ADHD clinical symptoms and caregiver HRQoL (MD = − 0.099, 95% CI − 0.140 to − 0.057). When adjusted for child and family factors, the association between ADHD symptoms and caregivers’ HRQoL was not statistically significant (MD = − 0.033, 95% CI − 0.080 to 0.014) (Table 7).

Compared to caregivers of children without ADHD, caregivers of children with ADHD had better HRQoL scores in the coping (MD = 0.069, 95% CI 0.026 to 0.112), independent living (MD = 0.032, 95% CI 0.004 to 0.060) and mental health (MD = 0.056, 95% CI 0.016 to 0.097) dimensions. When we include caregiver mental health in the adjusted analyses, there is no significant difference in HRQoL among caregivers of children with parent-reported ADHD (MD = 0.049, 95% CI − 0.008 to 0.107) or ADHD symptoms (MD = − 0.039, 95% CI − 0.082 to 0.003) (Tables 8 and 9), but only a positive association with the coping dimension (MD =  0.051, 95% CI 0.010 to 0.093).

## Association between child ADHD/ADHD symptoms and caregivers’ mental health

Considering child and family factors, caregivers of children with ADHD had poorer psychosocial problems than those of children without ADHD (MD = − 1.310, 95% CI − 2.439 to − 0.181) (Table 4). However, we did not observe a significant difference in caregivers’ mental health problems between children with and without ADHD clinical symptoms (Table 10).

**Table 4:** Association between caregivers’ mental health and child ADHD

Factors	Unadjusted analysis		Adjusted analysis	
	Mean difference	95% CI	Mean difference	95% CI
Child ADHD	0.866	− 0.302,2.034	− **1.310**	− **2.439,** − **0.181**
Child total difficulties			**0.166**	**0.123,0.209**
Child’s other medical conditions			− 0.100	− 0.527,0.328
Financial hardship			0.415	− 0.019,0.849
Caregiver relationship quality			− **1.139**	− **1.436,** − **0.841**
*Higher education, post-graduate as reference*				
Graduate diploma/Certificate			− 0.313	− 0.903,0.277
Bachelor degree			− 0.332	− 0.892,0.229
Advanced/diploma, certificate or other			0.096	− 0.458,0.651

SD: Standard deviation; CI: confidence interval; ADHD: Attention Deficit ;Hyperactivity Disorder; Significance level at 5%; Statistically significant estimates in bold; N = 1381

## Factors associated with caregivers’ HRQoL

Caregivers’ HRQoL were lower in those with financial hardship (MD = − 0.025, 95% CI − 0.045 to − 0.004), or with children with higher total difficulty scores (MD = − 0.005, 95% CI − 0.007 to − 0.003). Caregivers who completed a graduate diploma/certificate or diploma/certificate had lower HRQoL than those who completed post-graduate education. Caregiver relationship quality was associated with better caregivers’ HRQoL (MD = 0.027, 95% CI 0.016 to 0.016) (Table 3).

## Discussion

The inconsistent association between child ADHD and caregiver HRQoL varied across models, suggesting that it is not a direct or robust relationship but one that is strongly conditioned by contextual factors, especially caregiver mental health. The positive association between parent-reported child ADHD and caregiver HRQoL when considering child and family factors should be interpreted cautiously. It may be explained by the contextual factors, such as the high proportion of children in our sample (85%) receiving ADHD medication or parental coping as reflected in the positive association with the coping dimension. Child behavioural problems and parental stress may have bidirectional influences, whereby the improvement of ADHD symptoms may lower caregiver stress and have a positive effect on their HRQoL [57]. Research has shown that caregivers of children with ADHD who took medication had improved functional and HRQoL outcomes [57, 58]. However, due to the strong correlation with child social-emotional difficulties, ADHD medication was excluded from the analysis, limiting its causal interpretation.

The null association between parent-reported child ADHD and caregiver HRQoL once caregiver mental health was accounted for suggests that psychological distress may be a key pathway linking child ADHD to caregiver well-being [20, 21], rather than broad impairments across HRQoL domains. Conversely, while the negative association between ADHD symptoms and caregiver HRQoL observed in our unadjusted analysis somewhat aligns with the limited literature supporting this association for caregivers of children with ADHD symptoms, conducted in Asian countries and Spain [27, 59–61], the absence of a significant association when considering child and family factors in our study suggests the association is confounded by child and family factors.

We found that child ADHD were associated with better coping skills, mental health and independent living dimensions, but only the coping dimension remained associated with ADHD after accounting for caregiver mental health. This finding suggests that the effects of mental health and independent living dimensions largely reflect psychological distress, whereas coping may represent adaptive responses to caregiving demands. This finding should be interpreted cautiously. It may be that caregivers experienced heightened resilience or adaptive strategies to handle their situation in the presence of positive influences, e.g., perceived family or social support, positive parental strategies, improved parent–child relationships. Given the cross-sectional design, it is not possible to determine whether our finding reflects genuine adaptive coping strategies developed through ongoing exposure to caregiving challenges over time, selection effects (e.g. caregivers with greater coping capacity being more likely to remain in the study), or characteristics of the coping measure itself. Prior research indicates that caregivers of children with ADHD commonly utilise problem-focused coping, emotional regulation, and social support-seeking strategies, which are associated with better mental health outcomes and resilience [23] and had higher HRQoL, but many may struggle and have poor HRQoL. However, these mechanisms were not directly measured in the present study and therefore cannot be tested. Future longitudinal studies with direct measurement of coping strategies are needed to better understand the mechanisms underlying this finding.

Our finding reveals that child ADHD was associated with poorer caregiver mental health, whereas ADHD clinical symptoms were not. Similarly, the positive association between parent-reported child ADHD and caregiver HRQoL, which was fully attenuated after accounting for caregiver mental health, was not observed in caregivers of children with ADHD clinical symptoms. This likely reflects that those with ADHD may have more severe or functional challenges and additional caregiving stressors, including navigating services and managing comorbidities. While the SDQ hyperactivity-inattention subscale is a valid outcome measure for ADHD symptoms and has been used in many previous studies drawing data from LSAC [37, 64], it may underrepresent functional impact and be influenced by parent perception. Therefore, it is important to distinguish between ADHD diagnosis and symptom severity in understanding caregiver challenges in caring for a child with ADHD.

After adjusting for confounding factors, the results suggest that the association between child ADHD and caregiver HRQoL reflects a combination of multiple contributing factors, with each factor potentially modifying the observed relationship. Caregivers’ mental health problems, financial hardship or low level of education, child emotional behavioural problems may exacerbate the challenges associated with caring for a child with ADHD and thus are associated negatively with the HRQoL of these caregivers as observed in this study. This finding is consistent with previous literature reporting that child severity of emotional and ADHD symptoms, parental low level of education, low household income [59] and maternal depressive symptoms [27] were associated with poorer maternal HRQoL. Previous research also showed that high SDQ scores and externalising disorders were strongly correlated with poorer caregiver mental health outcomes [6, 7]. Understanding the factors that contribute to poor caregivers’ HRQoL is essential for evaluating and implementing interventions that improve HRQoL in both the caregiver and their children.

## Policy implications and future research directions

This study confirms patterns in caregiver mental health and HRQoL among caregivers of children with ADHD within a nationally representative Australian sample. Using multi-attribute utility-based measures, it provides population-level insight into caregiver well-being and extends prior findings beyond clinical or convenience samples.

Our finding suggests that while some caregivers of children with ADHD developed coping strategies and managed their HRQoL well, many may have struggled with their poor HRQoL and mental health. This finding indicates that caregivers of children with ADHD may benefit from a holistic approach to ADHD treatment and management. The last few decades have shown a push away from child-centred frameworks toward more holistic conceptual frameworks for managing services to children with disabilities or chronic conditions and their families [4]. Findings from our study provide insights into family-centred care that recognises the essential role of the family in the child’s life, recognises the insights into the child’s needs and abilities that parents can contribute, and places a greater emphasis on caregiver involvement [4, 7]. Our findings about the several factors, such as caregiver mental health, financial hardship and child comorbidities influencing caregivers’ HRQoL suggest that future ADHD interventions or supporting strategies could benefit from a family-centred approach that considers these factors, not just child ADHD. Given the cross-sectional nature of our study, future longitudinal studies are needed to understand the causal relationships between child ADHD and caregiver’s HRQoL, along with the mediation effect of caregiver mental health, as well as the long-term factors influencing caregiver’s HRQoL.

## Strengths and limitations

The strength of our study is the use of a nationally representative population-based sample, which strengthens the generalisability of the findings. The data were drawn from a study population that recruited children and their caregivers from birth prior to the identification of child health or behavioural issues associated with ADHD. This reduces participant selection bias.

Our study has some limitations of note. First, all measures included in the analysis were based on self-reports by the parents in the LSAC dataset, which are subject to single respondent bias. Second, there was no formal diagnosis for the child with ADHD, but just the parent report of the child’s ongoing ADHD. Furthermore, the reliance on parent self-report of the SDQ in identifying children with ADHD clinical symptoms may present some inaccuracy. However, the parent-reported SDQ hyperactivity-inattention subscale has strong internal consistency reliability (alpha coefficient of 0.74) [39]. While multi-informant data would enhance robustness, single-parent reporting remains highly informative, capturing symptom manifestation across settings, particularly the home environment where ADHD symptoms are most evident [65]. Previous research has also reported moderate-to-good concordance between parent and teacher reports of SDQ [66]. In addition, there may be children in the LSAC dataset who have ADHD but have not been diagnosed and were not included in this analysis. Third, the caregivers’ HRQoL data were only available from the Child Health Checkpoint dataset (about 50% of the Wave 6 respondents). The cross-sectional analysis precludes any inferences about the causal impact of caring for a child with ADHD on caregivers’ HRQoL. Furthermore, to avoid over-adjustment by conditioning on constructs that are closely aligned with, or partially constitutive of, the outcome, we excluded highly collinear variables such as the hyperactivity-inattention subscale of the SDQ, parental coping, efficacy, happiness, and mental health. This means the models reflect broader associations rather than the independent effects of overlapping psychosocial constructs. Future research using longitudinal or structural modelling approaches could better disentangle these interrelated pathways. We did not apply formal multiple-comparison adjustments because HRQoL dimensions are conceptually and empirically correlated, and our primary inference focused on overall HRQoL rather than individual domains. This might increase the risk of type I error, and thus, our findings about the coping dimension should be interpreted cautiously. However, conservative corrections (e.g. Bonferroni) may inflate type II error and mask potentially meaningful patterns [67]. Complete case analysis assumes data are missing completely at random, a stricter assumption than the missing at random assumption underlying multiple imputation [50]. However, the multiple imputation sensitivity analysis mirrored our findings, confirming their robustness. Finally, the small number of ADHD cases may result in unstable estimates with limited precision, such that effect sizes could vary substantially with small changes in the data and should therefore be interpreted cautiously.

## Conclusion

Child ADHD was associated with poor caregivers’ mental health, but not with overall HRQoL after accounting for caregiver mental health. Caregiver mental health, child socio-emotional behavioural issues, and financial hardship were associated with poorer caregiver HRQoL, while caregiver relationship quality was associated with better HRQoL. Given these cross-sectional associations, future longitudinal research is needed to establish temporal relationships. Health policies and strategies for families of children with ADHD may benefit from considering caregiver mental health and wellbeing with both holistic family-based approaches and targeted caregiver supports.

## Appendix

See Tables 5, 6, 7, 8, 9, 10 and 11.

This study explored the association between child ADHD and ADHD clinical symptoms and caregivers’ HRQoL/mental health, and the factors predicting caregivers’ HRQoL. We found that child ADHD was associated with poorer caregivers’ mental health, with no consistent evidence of better or lower overall HRQoL once caregiver mental health was considered. Conversely, child ADHD clinical symptoms were not associated with caregivers’ mental health or HRQoL.

**Table 5:** Description of selected variables used in the analyses

Variable	Measurement tool	Data value structure	Timepoint
*Outcome variables*			
Caregiver’s HRQoL	AQoL-8D	Continuous	Checkpoint
*Main predictor*			
Child ADHD	Parent report of child ADHD or ADD	Binary, yes/no	Wave 6
*Covariates*			
Study child sex	Parent report on child sex	Dichotomous, male/female	Wave 6
ADHD medication	Parent report if child is taking ADHD medication	Binary, yes/no	Wave 6
Child total difficulties	SDQ	Continuous, parent report	Wave 6
ADHD clinical symptoms	5-item hyperactivity subscale of the SDQ	Continuous, parent report	Wave 6
Other medical conditions	Parent report on whether child has an ongoing health condition. Composite variable combining eczema, asthma, vision problems, hearing problems, frequent headaches, diabetes, epilepsy, and chronic fatigue	Binary, yes/no	Wave 6
Parent sex	Parent self-report sex	Dichotomous, male/female	Wave 6
Parent age	Parent self-report age		Checkpoint
Caregiver depression	Kessler 6 depression scale	Continuous	Wave 6
Relationship quality	Hendrick relationship scale	Continuous	Wave 6
Socioeconomic difficulty	Financial hardship scale	Continuous	Wave 6
Higher education	Composite variable combining parent responses to questions whether they completed secondary school and the level of post-secondary education achieved	Categorical, (1) no post-secondary education, (2) certificate, advanced diploma, other, (3) bachelor degree (4) postgraduate degree or graduate diploma/certificate	Wave 6

HRQoL: Health-related quality of life; AQoL-8D: Assessment of Quality of Life-8 Dimensions; ADHD: Attention-deficity/hyperactivity disorder; ADD: Attention-deficit disorder; SDQ: Strengths and difficulties questionnaire

**Table 6:** Selected characteristics of children with and without ADHD clinical symptoms at 10–11 years

Characteristics	Study Child has ADHD clinical symptoms		
	No	Yes	Total
	N = 1745^a^	N = 109	N = 1854
Male, n (%)	823 (50.5%)	71 (65.1%)	895 (51.4%)
Child SDQ total score, mean (SD)	6.924 (4.760)	18.326 (5.638)	7.642 (5.558)
Child has ongoing ADHD	27 (1.6%)	35 (31.8%)	62 (3.5%)
Child had other medical conditions	575 (35.3%)	58 (52.5%)	633 (36.4%)
Child taking ADHD medication	14 (78.2%)	25 (22.9%)	39 (84.9%)
Parent 1 age, mean (SD)	42.095 (5.233)	42.842 (5.070)	42.142 (5.225)
Parent 1 depression scale score, mean (SD)	9.211 (3.467)	10.659 (4.215)	9.302 (3.535)
Hendrick relationship quality scale, mean (SD)	4.259 (0.762)	4.170 (0.803)	4.255 (0.764)
Hardship scale, mean (SD)	0.253 (0.672)	0.447 (0.972)	0.265 (0.696)
*Parent ‘s highest education level*			
Postgraduate degree	172 (12.3%)	8 (9.0%)	180 (12.1%)
Graduate diploma/certificate	153 (11.0%)	5 (5.5%)	158 (10.6%)
Bachelor degree	339 (24.3%)	17 (18.7%)	357 (24.0%)
Advanced diploma/diploma, certificate or other	730 (52.3%)	62 (66.8%)	792 (53.2%)

Continuous variables: Mean (SD); Categorical variables: n (%); Statistics computed using the checkpoint survey weights; SDQ: Strengths and difficulties questionnaire; parent response

**Table 7:** HRQoL in caregivers of children with ADHD symptoms

	Overall utility score		Coping		Happiness		Independent living		Mental health		Pain		Relationship		Senses		Self-worth	
Child ADHD symptoms	− 0.033	− 0.080,0.014	− 0.011	− 0.045,0.023	− 0.032	− 0.068,0.005	− 0.009	− 0.033,0.016	− 0.014	− 0.047,0.019	− 0.034	− 0.101,0.033	− 0.025	− 0.064,0.014	− 0.010	− 0.036,0.017	− 0.016	− 0.053,0.021
Internalising problems	**0.050**	**0.037,0.062**	**0.025**	**0.015,0.036**	**0.036**	**0.027,0.045**	**0.012**	**0.004,0.019**	**0.036**	**0.027,0.044**	**0.017**	**0.001,0.032**	**0.057**	**0.046,0.069**	**0.016**	**0.007,0.025**	**0.027**	0.018,0.037
Externalising problems	− **0.015**	− **0.020,**− **0.010**	− **0.012**	− **0.016,**− **0.008**	− **0.007**	− **0.010,**− **0.004**	− **0.004**	− **0.007,**− **0.001**	− **0.010**	− **0.013,**− **0.006**	− **0.007**	− **0.013,**− **0.000**	− **0.012**	− **0.016,**− **0.008**	− **0.005**	− **0.008,-0.001**	− **0.012**	− 0.016,-0.008
Other medical conditions	− **0.011**	− **0.019,**− **0.004**	− **0.006**	− **0.012,**− **0.000**	− 0.004	− 0.009,0.001	− 0.002	− 0.007,0.002	− **0.010**	− **0.015,**− **0.004**	− **0.013**	− **0.023,**− **0.003**	− **0.007**	− **0.013,**− **0.001**	− 0.002	− 0.007,0.003	− **0.006**	− **0.012,**− **0.001**
Caregiver relationship quality	− 0.013	− 0.030,0.005	− 0.006	− 0.020,0.008	0.001	− 0.011,0.013	− 0.011	− 0.021,0.000	0.003	− 0.011,0.017	− **0.026**	− **0.049,**− **0.004**	− 0.012	− 0.027,0.002	− 0.012	− 0.025,0.001	− 0.007	− 0.022,0.008
Financial hardship	− **0.024**	− **0.044,**− **0.003**	− **0.017**	− **0.034,**− **0.000**	− 0.012	− 0.029,0.004	− **0.015**	− **0.028,**− **0.002**	− **0.018**	− **0.029,**− **0.007**	− 0.021	− 0.044,0.002	− 0.012	− 0.031,0.007	− 0.010	− 0.026,0.007	− 0.009	− 0.022,0.004
Higher education, post-graduate as reference																		
Graduate diploma/Certificate	− 0.019	− 0.045,0.006	− 0.003	− 0.023,0.016	− 0.010	− 0.027,0.007	− 0.005	− 0.020,0.011	− 0.007	− 0.032,0.017	− 0.014	− 0.050,0.022	− 0.006	− 0.033,0.020	− **0.026**	− **0.049,**− **0.003**	− 0.020	− 0.041,0.000
Bachelor degree	0.008	− 0.011,0.027	0.003	− 0.013,0.019	− 0.006	− 0.020,0.009	0.002	− 0.008,0.013	0.008	− 0.010,0.026	0.019	− 0.010,0.047	0.016	− 0.005,0.037	− 0.001	− 0.018,0.016	− 0.004	− 0.019,0.011
Advanced/diploma, certificate or other	− **0.026**	− **0.046,**− **0.007**	− 0.011	− 0.027,0.005	− 0.009	− 0.022,0.005	− **0.017**	− **0.030,**− **0.004**	− 0.003	− 0.021,0.015	− **0.040**	− **0.067,**− **0.013**	− 0.006	− 0.026,0.014	− 0.011	− 0.028,0.007	− **0.039**	− **0.055,**− **0.022**

SD: Standard deviation; CI: confidence interval; ADHD: Attention Deficit Hyperactivity Disorder; HRQoL: Health-related quality of life; Significance level at 5%; Statistically significant estimates in bold.; N = 1855.

**Table 8:** HRQoL in caregivers of children with ADHD

Factors	Overall utility scores		Coping dimension		Happiness dimension		Independent living dimension		Mental health dimension		Pain dimension		Relationship dimension		Sense dimension		Self-worth dimension	
	**Mean**	**95% CI**	**Mean**	**95% CI**	**Mean**	**95% CI**	**Mean**	**95% CI**	**Mean**	**95% CI**	**Mean**	**95% CI**	**Mean**	**95% CI**	**Mean**	**95% CI**	**Mean**	**95% CI**
Child ADHD	0.049	− 0.008,0.107	**0.051**	**0.010,0.093**	0.015	− 0.037,0.067	0.022	− 0.007,0.052	0.038	− 0.001,0.077	0.037	− 0.024,0.098	0.017	− 0.040,0.074	0.005	− 0.033,0.043	0.033	− 0.020,0.086
Caregiver mental heath	− **0.020**	− **0.023,** − **0.016**	− **0.013**	− **0.016,** − **0.011**	− **0.011**	− **0.013,** − **0.009**	− **0.007**	− **0.010,** − **0.004**	− **0.014**	− **0.016,** − **0.012**	− **0.013**	− **0.018,** − **0.007**	− **0.013**	− **0.015,** − **0.010**	− **0.004**	− **0.007,** − **0.000**	− **0.016**	− **0.020,** − **0.013**
Caregiver relationship quality	**0.027**	**0.016,0.039**	**0.010**	**0.000,0.020**	**0.023**	**0.014,0.031**	0.003	− 0.003,0.010	**0.020**	**0.012,0.028**	0.002	− 0.013,0.018	**0.042**	**0.031,0.054**	**0.012**	**0.003,0.021**	0.008	− 0.000,0.017
SDQ total score	− **0.005**	− **0.007,** − **0.003**	− **0.004**	− **0.005,** − **0.002**	− **0.002**	− **0.003,** − **0.001**	− 0.001	− 0.002,0.000	− **0.003**	− **0.005,** − **0.002**	− **0.004**	− **0.007,** − **0.001**	− **0.004**	− **0.005,** − **0.002**	− 0.001	− 0.003,0.000	− **0.003**	− **0.005,** − **0.002**
Financial hardship	− 0.016	− 0.036,0.004	− 0.012	− 0.028,0.005	− 0.008	− 0.024,0.008	− 0.012	− 0.024,0.000	− **0.013**	− **0.023,** − **0.002**	− 0.016	− 0.038,0.006	− 0.007	− 0.026,0.012	− 0.008	− 0.026,0.009	− 0.003	− 0.015,0.009
Other medical conditions	− **0.015**	− **0.030,** − **0.001**	− 0.008	− 0.020,0.004	− 0.001	− 0.012,0.010	− **0.011**	− **0.022,** − **0.001**	0.001	− 0.010,0.013	− **0.027**	− **0.048,** − **0.005**	− **0.014**	− **0.028,** − **0.001**	− 0.013	− 0.026,0.000	− 0.009	− 0.021,0.003
*Higher education, post-graduate as reference*																		
Graduate diploma/Certificate	− **0.031**	− **0.053,** − **0.009**	− 0.013	− 0.031,0.005	− **0.017**	− **0.033,** − **0.001**	− 0.009	− 0.024,0.006	− 0.015	− 0.038,0.007	− 0.018	− 0.053,0.017	− 0.015	− 0.039,0.008	− **0.030**	− **0.052,** − **0.007**	− **0.031**	− **0.050,** − **0.012**
Bachelor degree	0.001	− 0.017,0.017	− 0.003	− 0.018,0.012	− 0.01	− 0.024,0.003	− 0.001	− 0.011,0.010	0.002	− 0.015,0.019	0.014	− 0.015,0.043	0.01	− 0.009,0.029	− 0.002	− 0.020,0.015	− 0.011	− 0.026,0.003
Advanced/diploma, certificate or other	− **0.025**	− **0.041,** − **0.009**	− 0.011	− 0.024,0.003	− 0.008	− 0.020,0.004	− **0.016**	− **0.029,** − **0.004**	− 0.002	− 0.018,0.013	− **0.038**	− **0.064,-0.011**	− 0.005	− 0.023,0.013	− 0.011	− 0.028,0.007	− **0.038**	− **0.053,** − **0.023**

SD: Standard deviation; CI: confidence interval; ADHD: Attention Deficit Hyperactivity Disorder; HRQoL: Health-related quality of life; Significance level at 5%; Statistically significant estimates in bold; N = 1855.

**Table 9:** HRQoL in caregivers of children with ADHD symptoms

Child ADHD symptoms	Overall utility score		Coping		Happiness		Independent living		Mental health		Pain		Relationship		Senses		Self-worth	
	− 0.039	− 0.082,0.003	− 0.015	− 0.048,0.017	− 0.035	− 0.071,0.001	− 0.011	− 0.034,0.013	− 0.019	− 0.049,0.011	− 0.038	− 0.101,0.025	− 0.029	− 0.069,0.011	− 0.011	− 0.038,0.016	− 0.021	− 0.055,0.013
Internalising problems	− **0.008**	− **0.012,**− **0.004**	− **0.007**	− **0.011,**− **0.004**	− **0.003**	− **0.006,**− **0.001**	− 0.002	− 0.004,0.001	− **0.005**	− **0.008,**− **0.001**	− 0.002	− 0.008,0.005	− **0.008**	− **0.011,**− **0.004**	− 0.003	− 0.007,0.000	− **0.006**	− **0.010,**− **0.003**
Externalising problems	− **0.007**	− **0.013,**− **0.001**	− 0.003	− 0.009,0.002	− 0.001	− 0.006,0.003	− 0.001	− 0.005,0.003	− **0.006**	− **0.011,**− **0.002**	− 0.01	− 0.019,0.000	− 0.004	− 0.009,0.001	− 0.001	− 0.006,0.004	− 0.002	− 0.007,0.003
Other medical conditions	− 0.015	− 0.029,0.000	− 0.007	− 0.019,0.005	0	− 0.011,0.011	− **0.011**	− **0.021,**− **0.001**	0.002	− 0.010,0.014	− **0.027**	− **0.049,**− **0.006**	− 0.014	− 0.027,0.000	− 0.012	− 0.025,0.001	− 0.008	− 0.020,0.004
Caregiver relationship quality	**0.027**	**0.016,0.039**	**0.010**	**0.000,0.020**	**0.023**	**0.014,0.031**	0.003	− 0.003,0.010	**0.020**	**0.012,0.028**	0.002	− 0.013,0.017	**0.043**	**0.031,0.054**	**0.012**	**0.003,0.021**	**0.009**	**0.000,0.017**
Caregiver mental heath	− **0.020**	− **0.023,**− **0.017**	− **0.013**	− **0.016,**− **0.011**	− **0.011**	− **0.013,**− **0.009**	− **0.007**	− **0.011,**− **0.004**	− **0.014**	− **0.016,**− **0.012**	− **0.013**	− **0.018,**− **0.008**	− **0.013**	− **0.015,**− **0.011**	− **0.004**	− 0.007,− 0.000	− **0.016**	− **0.020,**− **0.013**
Financial hardship	− 0.015	− 0.035,0.005	− 0.011	− 0.028,0.005	− 0.008	− 0.024,0.008	− 0.012	− 0.024,0.000	− **0.012**	− **0.023,**− **0.001**	− 0.015	− 0.038,0.007	− 0.007	− 0.026,0.012	− 0.008	− 0.025,0.009	− 0.002	− 0.014,0.010
*Higher education, post-graduate as ref*																		
Graduate diploma/Certificate	− **0.029**	− **0.052,**− **0.006**	− 0.01	− 0.029,0.009	− 0.016	− 0.032,0.000	− 0.008	− 0.024,0.007	− 0.014	− 0.037,0.009	− 0.02	− 0.056,0.015	− 0.013	− 0.037,0.011	− **0.028**	− **0.051,**− **0.005**	− **0.028**	− **0.047,**− **0.010**
Bachelor degree	0.001	− 0.016,0.018	− 0.002	− 0.017,0.013	− 0.010	− 0.023,0.003	− 0.001	− 0.011,0.010	0.003	− 0.014,0.019	0.013	− 0.015,0.042	0.011	− 0.008,0.030	− 0.002	− 0.019,0.015	− 0.011	− 0.025,0.004
Advanced/diploma, certificate or other	− **0.025**	− **0.041,**− **0.008**	− 0.01	− 0.024,0.004	− 0.008	− 0.020,0.005	− **0.016**	− **0.028,**− **0.004**	− 0.002	− 0.018,0.013	− **0.039**	− **0.065,**− **0.013**	− 0.005	− 0.023,0.013	− 0.01	− 0.028,0.007	− **0.038**	− **0.052,**− **0.023**

SD: Standard deviation; CI: confidence interval; ADHD: Attention Deficit Hyperactivity Disorder; HRQoL: Health-related quality of life; Significance level at 5%; Statistically significant estimates in bold; N = 1855.

**Table 10:** Mental health in caregivers of children with ADHD symptoms

Factors	Unadjusted analysis		Adjusted analysis	
	Mean difference	95% CI	Mean difference	95% CI
Child ADHD symptoms	**1.451**	**0.507, 2.396**	− 0.315	− 1.298,0.669
Internalising problems			**0.355**	**0.241,0.468**
Externalising problems			**0.234**	**0.039,0.429**
Other medical conditions			− 0.106	− 0.528,0.317
Caregiver relationship quality			− **1.143**	− **1.446,**− **0.839**
Financial hardship			0.407	− 0.026,0.840
*Higher education, post-graduate as a reference*				
Graduate diploma/Certificate			− 0.461	− 1.051,0.130
Bachelor degree			− 0.371	− 0.937,0.195
Advanced/diploma, certificate or other			0.105	− 0.448,0.658

SD: Standard deviation; CI: confidence interval; ADHD: Attention Deficit Hyperactivity Disorder; HRQoL: Health-related quality of life; Significance level at 5%; Statistically significant estimates in bold; N = 1855.

**Table 11:** STROBE Statement—checklist of items that should be included in reports of cross-sectional studies

	Item No	Recommendation
Title and abstract	1	(*a*) Indicate the study’s design with a commonly used term in the title or the abstract
		Pg1 at the cross-sectional Child Health CheckPoint data collection
		(*b*) Provide in the abstract an informative and balanced summary of what was done and what was found
		Pg1
*Introduction*		
Background/rationale	2	Explain the scientific background and rationale for the investigation being reported
		Pgs 2–3: Background on ADHD, HRQoL and stress and coping conceptual frameworks, caregiver stress and family functioning theoretical frameworks and the rationale for the research are presented
Objectives	3	State specific objectives, including any prespecified hypotheses
		Pg 3: (1) the association between child ADHD or /ADHD clinical symptoms and caregivers’ HRQoL; (2) which domains are most affected by child ADHD or ADHD symptoms; (3) association between child ADHD or ADHD symptoms and caregiver mental health; and (4) predictive factors influencing caregivers’ HRQoL
*Methods*		
Study design	4	Present key elements of study design early in the paper
		Pg 3: Child Health Checkpoint dataset was a one-off cross-sectional bio-physical data collection conducted between waves 6 and 7 for consenting primary caregivers (parent 1) and their children from the B cohort at 11 to 12 years old/wave 6.5 (N = 1,874)
Setting	5	Describe the setting, locations, and relevant dates, including periods of recruitment, exposure, follow-up, and data collection
		Pg3: Australian children and caregivers; LSAC commencement in 2004, biennial data collection
Participants	6	(*a*) Give the eligibility criteria, and the sources and methods of selection of participants
		Pg 3: Child Health Checkpoint dataset B cohort at 11 to 12 years old (N = 1,874); Study participants were both the child and their caregivers; rationale given for sourcing relevant variables from LSAC Wave 6 (age 10–11; n = 3,764). These data were linked for analysis
Variables	7	Clearly define all outcomes, exposures, predictors, potential confounders, and effect modifiers. Give diagnostic criteria, if applicable
		Pgs 4–5: The Methods, Measures and Statistical analysis section provide details on the outcomes, predictors and the criteria for covariates to be selected in the analysis
Data sources/ measurement	8*	For each variable of interest, give sources of data and details of methods of assessment (measurement). Describe comparability of assessment methods if there is more than one group
		Pg 4–5: We clearly state how we chose these covariates theoretically and then employ a data-driven method to refine these covariates. For example:
		“Covariates were first informed by the stress-process and family systems frameworks [5] and prior evidence [20, 21] and refined using statistical criteria to ensure model parsimony and stability. Multicollinearity was assessed using Pearson correlations, and highly collinear variables were not included simultaneously. The cutoff for multicollinearity coefficients was determined at > 0.5 due to the potential impact on the study results [53]. Six variables were excluded due to moderate (> 0.5) to strong (≥ 0.7) correlation: hyperactivity-inattention subscale, caregiver mental health, taking ADHD medication, ‘happiness in relationship’, parental efficacy, and parental coping. “
Bias	9	Describe any efforts to address potential sources of bias
		Pg 5 statistical analysis, addressing sample size, missing data and covariates, for example:” The CheckPoint sample weight was used to correct for sample disproportionality (e.g. slightly more advantaged and less likely to be from regional or remote area, an Indigenous or non-English speaking background than LSAC wave 1) and to compensate for attrition rates (37%) and nonresponse bias”
		Pg 9 discussion: limitations
		“While the SDQ hyperactivity-inattention subscale is a valid outcome measure for ADHD symptoms and has been used in many previous studies drawing data from LSAC [38, 74], it may underrepresent functional impact and be influenced by parent perception.”
		“all measures included in the analysis were based on self-reports by the parents in the LSAC dataset, which are subject to single respondent bias”
		“the small number of ADHD cases may result in unstable estimates with limited precision”
Study size	10	Explain how the study size was arrived at
		Pg 5: Statistical analysis; “The LSAC wave 6, B cohort dataset and the Child Health CheckPoint datasets (wave 6.5) were merged according to participant identifier number. There were 1,874 total matched observations in the analytic sample. Nineteen participants in the sample were missing data identifying their children’s ADHD status (n = 6) or HRQoL data (n = 13) and were excluded from the analysis.”
Quantitative variables	11	Explain how quantitative variables were handled in the analyses. If applicable, describe which groupings were chosen and why
		Pg 3–4 Methods section: explanation of different quantitative variables and groupings; Pg 5: statistical analysis section
Statistical methods	12	(*a*) Describe all statistical methods, including those used to control for confounding
		P5: covariates informed by the stress-process and family systems frameworks and prior evidence and refined using statistical criteria to ensure model parsimony and stability. Multicollinearity was assessed using variance inflation factors and Pearson correlations. Rationale for cut-off score was given
		(*b*) Describe any methods used to examine subgroups and interactions
		P5: The statistical analyses section provide details on the analysis
		Primary analysis focuses on the association between child ADHD and caregiver HRQoL, excluding caregiver mental health. Given its central role in the stress-process and family systems frameworks, caregiver mental health was examined separately in a sensitivity analysis to explore the extent to which observed HRQoL differences were statistically associated with caregiver psychological distress
		A secondary analysis was conducted to explore the association between ADHD clinical symptoms and caregivers’ HRQoL using the same set of covariates
		(*c*) Explain how missing data were addressed
		Pg 5: Nineteen participants in the sample were missing data identifying their children’s ADHD status (N = 6) or HRQoL data (N = 13) and were excluded from the analysis. Due to a small amount of missing data (10%), multiple imputation is not recommended
		(*d*) If applicable, describe analytical methods taking account of sampling strategy
		Pg 5 The checkpoint sample weight was used to correct for sample disproportionality and to compensate for attrition rates and nonresponse bias
		(*e*) Describe any sensitivity analyses
		Pg 5, included in revision “We included caregiver mental health in a sensitivity analysis…”
*Results*		
Participants	13*	(a) Report numbers of individuals at each stage of study—eg numbers potentially eligible, examined for eligibility, confirmed eligible, included in the study, completing follow-up, and analysed
		Pg 6, Results, and In Table 1 and 6
		(b) Give reasons for non-participation at each stage
		NA
		(c) Consider use of a flow diagram
		NA
Descriptive data	14*	(a) Give characteristics of study participants (e.g., demographic, clinical, social) and information on exposures and potential confounders
		Table 1, selected child and family characteristics and pg 5–6
		(b) Indicate number of participants with missing data for each variable of interest
		P5: as above
Outcome data	15*	Report numbers of outcome events or summary measures
		Pg 5–6
Main results	16	(*a*) Give unadjusted estimates and, if applicable, confounder-adjusted estimates and their precision (eg, 95% confidence interval). Make clear which confounders were adjusted for and why they were included
		Pg 6–7: for example, we reported:
		In unadjusted analyses, we did not observe a statistically significant association between child ADHD and overall caregivers’ HRQoL at children aged 11–12 years (mean difference [MD] = -0.031, 95% CI -0.008 to 0.026) (Table 2). When adjusted for child and family factors, we observed a positive association between parent-reported child ADHD and their HRQoL (MD = 0.076, 95% CI 0.026 to 0.112) (Table 3)
		Association between child ADHD/ADHD symptoms and caregivers’ HRQoL/HRQoL dimensions;
		Factors associated with caregivers’ HRQoL
		(*b*) Report category boundaries when continuous variables were categorized
		NA
		(*c*) If relevant, consider translating estimates of relative risk into absolute risk for a meaningful time period
		NA
Other analyses	17	Report other analyses done—eg analyses of subgroups and interactions, and sensitivity analyses
		P5: Association between child ADHD/ADHD symptoms and caregivers’ mental health
		Analysis including caregiver mental health
*Discussion*		
Key results	18	Summarise key results with reference to study objectives
		Pg 6: we present study results reference to study objectives: association between child ADHD/ADHD symptoms and caregiver HRQoL; association between child ADHD/ADHD symptoms and caregiver mental health and factors influencing caregiver HRQoL
		Pg 7: we also summary our study finding in Discussion
Limitations	19	Discuss limitations of the study, taking into account sources of potential bias or imprecision. Discuss both direction and magnitude of any potential bias
		Pg 9: we provided a list of possible limitations. For example,
		“First, all measures included in the analysis were based on self-reports by the parents in the LSAC dataset, which are subject to single respondent bias. Second, there was no formal diagnosis for the child with ADHD, but just the parent report of the child's ongoing ADHD. Furthermore, the reliance on parent self-report of the SDQ in identifying children with ADHD clinical symptoms may present some inaccuracy. However, the parent-reported SDQ hyperactivity-inattention subscale has strong internal consistency reliability (alpha coefficient of 0.74) “
Interpretation ”	20	Give a cautious overall interpretation of results considering objectives, limitations, multiplicity of analyses, results from similar studies, and other relevant evidence
		Pgs 9–10: we discussed overall study findings considering our study aims, results and relevant literature. For example: “This study contributes by confirming patterns in caregiver mental health and HRQoL … within a nationally representative Australian sample [and]… provides population-level insight into caregiver well-being and highlights the relevance of these patterns beyond clinical or convenience samples
Generalisability	21	Discuss the generalisability (external validity) of the study results
		Primarily addressed in limitations, pg 9: for example:
		“the small number of ADHD cases may result in unstable estimates with limited precision, such that effect sizes could vary substantially with small changes in the data and should therefore be interpreted cautiously.”
		Pg 10:
		Recommendations for future longitudinal research to determine temporal relationships and recommendations for health strategies and policies that consider caregiver HRQoL in families of children with ADHD with both holistic family-based approaches and targeted caregiver-focused support
*Other information*		
Funding	22	Give the source of funding and the role of the funders for the present study and, if applicable, for the original study on which the present article is based
		pg 19: Statement of funding, competing interests, author contributions

*Give information separately for exposed and unexposed groups. An Explanation and Elaboration article discusses each checklist item and gives methodological background and published examples of transparent reporting. The STROBE checklist is best used in conjunction with this article (freely available on the Web sites of PLoS Medicine at http://www.plosmedicine.org/, Annals of Internal Medicine at http://www.annals.org/, and Epidemiology at http://www.epidem.com/). Information on the STROBE Initiative is available at www.strobe-statement.org

## Data Availability

The Australian Institute for Family Studies approved the data used in this paper. The authors do not have permission to share the data. Data requests should be made following the LSAC public available form and appropriate approval would be needed.
